# Pressure Gradient-Driven Embolization b-TACE for HCC: Technical and Diagnostic Step-by-Step Procedural Guide and Literature Review

**DOI:** 10.3390/diagnostics15131726

**Published:** 2025-07-07

**Authors:** Bianca Rocco, David C. Madoff, Fabrizio Basilico, Elio Damato, Paolo Vetri, Valeria Panebianco, Carlo Catalano, Pierleone Lucatelli

**Affiliations:** 1 Interventional Radiology UOS, Department of Diagnostic Medicine and Radiology, Sapienza University of Rome, 00161 Rome, Italy; biancarocco.br@gmail.com (B.R.); fabriziobasilico.fb@gmail.com (F.B.); damato.elio91@gmail.com (E.D.); paolovvetri@gmail.com (P.V.); valeria.panebianco@uniroma1.it (V.P.); carlo.catalano@uniroma1.it (C.C.); 2 Interventional Radiology UOSD, Azienda Ospedaliera San Giovanni Addolorata, 00184 Rome, Italy; 3 Department of Radiology and Biomedical Imaging and Department of Internal Medicine (Medical Oncology), Yale School of Medicine, New Haven, CT 06520, USA; david.madoff@yale.edu

**Keywords:** hepatocellular carcinoma, transarterial chemoembolization, microballoon interventions

## Abstract

*Background:* Hepatocellular carcinoma (HCC) is one of the leading cause of cancer death worldwide. Transarterial therapies represent an important tool in the management of different clinical scenarios, from a patient with a single nodule to a patient with multinodular disease. Up to 30% of patients are diagnosed with intermediate-stage HCC, and transarterial chemoembolization (TACE) represents the mainstay of treatment. Overall survival in patients with HCC undergoing TACE is strongly influenced by obtaining a sustained complete response, which is strongly affected by the HCC’s dimension. *Methods:* Pressure gradient-driven embolization, achieved by employing a microballoon catheter in the balloon-occluded TACE (bTACE), represents the most novel innovation in the field of transarterial therapies in the last decade. In fact, bTACE, thanks to its ability to redistribute flow towards tumor territories, can allow higher chemotherapeutic drug concentrations, leading to better oncological performance, especially in patients in which standard TACE struggles to obtain a complete response. *Conclusions:* This technical and diagnostic intraprocedural step-by-step guide, discussed with a review of the existing literature, will enable readers to achieve an optimal procedure and to convey to their patients the full clinical benefits of these procedures.

## 1. Introduction

Primary liver cancer is the sixth most diagnosed cancer and the third leading cause of cancer death worldwide in 2020, with 830,000 deaths per year [1]. Hepatocellular carcinoma accounts for 75–85% of primary liver cancers, and its incidence rates have been constantly rising in the past decades [2]. It is projected to remain the third leading cause of cancer-related death by 2030 [3]. Staging HCC requires the consideration of more variables than the tumor burden: liver functionality and performance status have a central role in all staging systems. In 1999, the Barcelona Clinic Liver Cancer (BCLC) staging system [4] was proposed as a tool for both staging and treatment decisions, and in 2022, the last update was released [5].

According to the latest update of the BCLC system, trans-catheter therapies are pivotal in the management of HCC. Both transarterial chemoembolization (TACE) and transarterial radioembolization (TARE) are second-line strategies for patients at very early (0) and early stage (A), when curative treatments such as ablation or resection are not feasible [6].

Despite surveillance programs allowing HCC diagnosis in early stages in most patients, up to 30% of patients are diagnosed in the intermediate stage [7]. TACE has been globally adopted as the standard of care for a subgroup of patients with intermediate-stage HCC (in the early 2000s, two randomized trials and a meta-analysis recognized survival benefits in patients treated with TACE [8,9,10]), whenever liver transplantation is not feasible or the tumor is infiltrative or extensive. It is also important to highlight that in liver transplant candidates, transarterial strategies can be used for downstaging the disease as well as bridging whenever the waiting time for a transplant exceeds 6 months.

Materials employed during TACE to deliver chemotherapeutic drugs into the HCC tumor to occlude its arterial feeders include an emulsion of lipiodol and chemotherapeutic drugs in conventional TACE (c-TACE) and drug-eluting microspheres loaded with chemotherapeutic drugs (DEM) in DEM-TACE. Although DEM-TACE has demonstrated a better post-procedural pain profile in randomized trials and meta-analysis [10,11,12,13,14], the superior oncological results of one technique compared to another remains a subject of debate, and technique choice is left to the operator’s preference.

In TARE procedures, a tumoricidal dose of radiation is carried into the tumorous tissue. The radionuclide employed was yttrium-90—labeled to resin microspheres (SIR-Spheres) or embedded in glass microspheres (TheraSpheres)—or Holmium-166—labeled to microspheres (QuiremSpheres)-.

It is known from several studies that obtaining a complete response, according to the modified Response Evaluation Criteria in Solid Tumors (mRECIST) criteria [15], to the TACE procedure (or multiple TACE sessions) is the strongest positive predictor factor for a better overall survival (OS) prognosis [16,17], and a sustained response duration of 6 months or more has been found to have the strongest association with 5-year OS [18].

Achieving a complete response is known to be more challenging in tumors larger than 3 cm [19], as well as in tumors localized in non-peripheral segments (such as segments 1 and 4) [20,21,22]. This occurs because in central locations, intrahepatic collaterals and anastomosis, running between right and left hepatic arteries, are more common, affecting both the feeder’s selection and revascularization of the tumor after the treatment.

For these reasons and due to the variety of clinical scenarios in which transarterial therapies play a role in the management of patients with HCC, technical novelties have been proposed, aiming to ameliorate the therapeutic effect, and balloon occlusion has emerged as a strategy to improve the oncological responses.

## 2. Microballoon Interventions (Balloon-Occluded TACE -bTACE- and TARE -bTARE-): History and Rationale

Microballon catheters were employed for the first time by Nakamura in 1985 to prevent the embolization of gastric vessels [23] and by Irie in 1991 to perform a superselective balloon-occluded TACE (b-TACE), and in 2013, its mechanism and clinical implications started to be investigated [24]. This technique was applied for c-TACE first, and only for DEM-TACE until 2017 [25]. Regarding TARE, microballoons were used initially merely as antireflux devices [26], and in 2022, the first experiences for improving the oncological performance were reported [27].

The key to improved outcomes with balloon-occluded procedures is the possibility to redistribute flow towards the target lesion, consequently allowing the amount of chemotherapeutic drugs/radionuclide to concentrate within the tumor, preserving at the same time the surrounding parenchyma. The mechanism underlying flow restoration beyond the temporarily occluded segment is the opening of intrahepatic collaterals [28]. This compensatory process has been observed in in silico zero- and three-dimensional models of hepatic artery hemodynamics, mainly by Aramburu and colleagues [29,30,31,32].

**Figure 1:**
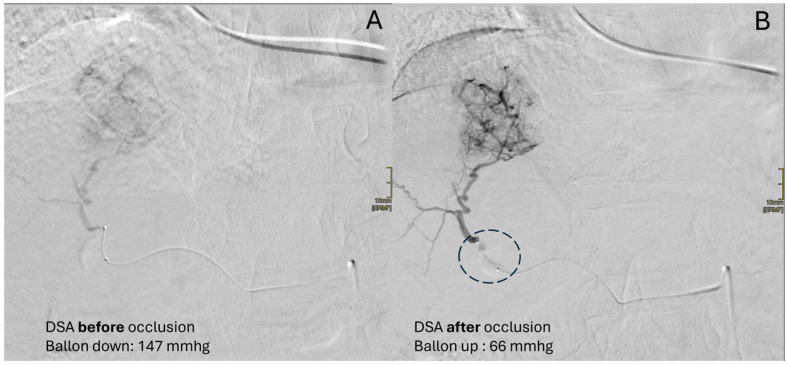
Clinical case of an 82-year-old female with HCC in HCV-related cirrhosis, treated with multiple locoregional treatments until 2022. In April 2024, an HCC nodule (sVII) up to 5 cm was newly diagnosed. The selective DSA performed with the deflated microballoon catheter (**A**) depicted the slightly hypervascular HCC, but not its microvasculature. The microballoon catheter’s inflation (dotted circle) due to flow redistribution improved HCC vascularization and the detection of its microvascularization, with a better subsequent diagnostic value of the DSA (**B**).

Another advantage of achieving flow redistribution thanks to microballoon catheters is the higher diagnostic performance of angiography performed in the occluded vessel, allowing the detection of less hypervascular tumors (Figure 1).

Demonstrations of flow redistribution in in vivo studies began in 2013, when Irie et al. demonstrated that when the balloon-occluded arterial stump pressure (BOASP), measured at the tip of the microcatheter with the balloon inflated, decreased to 64 mmHg or less, a deeper penetration of the chemotherapeutic drugs and embolization material within the tumor and its vessels was achieved. At the same time, the ratio of the lipidol emulsion concentration of HCC to embolized liver parenchyma was higher with respect to those treatments in which a BOASP of 64 mmHg was not obtained [24].

Following Irie, several authors demonstrated a significantly higher concentration of the chemotherapeutic drugs in the tumor due to balloon occlusion. Rose et al. compared the mean dose of drug or emulsion delivered in patients undergoing TACE with and without the “balloon-assisted technique” and found a significantly higher mean dose of drug or emulsion delivered using the bTACE procedure [28]. These results were confirmed by another study conducted by our group, evaluating the role of the balloon microcatheter in vivo in transarterial intervention comparing DEM-bTACE vs. DEM-TACE and bTARE vs. TARE [27]. Among the two DEM-TACE groups, the balloon microcatheter-treated groups showed a significantly higher contrast, signal-to-noise ratio, and contrast-to-noise ratio compared to conventional DEM-TACE, demonstrating a better profile in microparticle deposition within the tumor compared to the surrounding health parenchyma. In analyzing the two TARE groups, instead, the 2D dosimetry profile evaluation showed an activity intensity peak significantly higher in the bTARE than in the TARE subgroup. Moreover, through histological analysis in the explanted liver, there was a trend for higher intra-tumoral localization of embolic microspheres for DEM-bTACE in comparison with DEM-TACE. The results of this study demonstrate, in vivo, a better embolization profile of oncological intra-arterial interventions performed with a balloon microcatheter regardless of the embolic agent employed.

## 3. bTACE and bTARE: Safety and Oncological Results

bTACE safety has been widely demonstrated both in its application with lipiodol emulsion and with drug-eluting microspheres [33].

Already in 2016, Maruyama et al. [34] demonstrated c-bTACE safety, reporting liver abscess and infarction only in patients with bile duct dilatation (probably because of the access to peribiliary plexus, terminal arterio-sinus twigs and vasa vasorum permitted by the flow redistribution obtained with microballoon inflation). In this study, according to Irie’s previous experience, only the elevation of ALT was more frequent in the bTACE group, probably because this technique achieved a more severe ischemia.

Our group reported the first experience with DEM-bTACE [25]. In this series, adverse events were in line with the ones described for c-bTACE and DEM-TACE. One pseudo-aneurysm of the arterial feeder was reported, strictly related to the use of the balloon microcatheter, due to the learning curve for the correct use of this device. In the subsequent retrospective case–control study on DEM-bTACE vs. DEM-TACE [35], no significant differences were found in the incidence of adverse events or in laboratory value changes.

The oncological advantage of employing balloon occlusion has been demonstrated by several authors. Regarding its application with microspheres, DEM-bTACE has shown a trend for better oncological response over DEM-TACE, with a longer time to retreat interval, and a similar adverse event rate in patients presenting with larger tumors [35].

**Figure 2:**
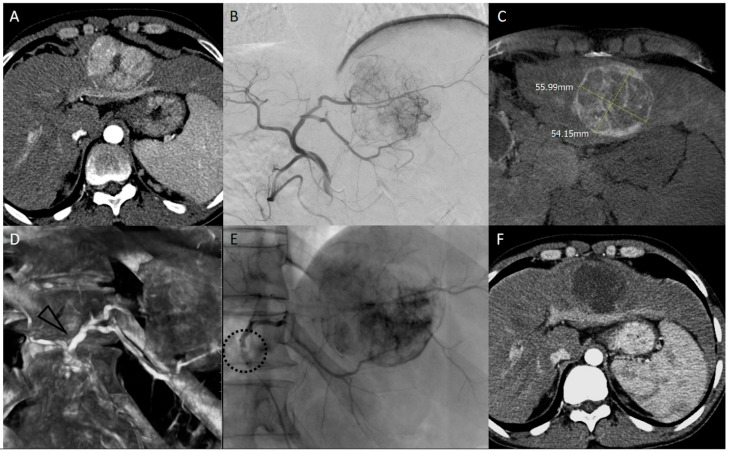
Clinical case of DEM-bTACE in a 41-year-old man with HBV-related cirrhosis, performed as bridging to orthotopic liver transplant. Pre-procedural arterial phase CT (**A**) showed a single 5.5 cm hepatocellular carcinoma in segment II-III. The transfemoral digital subtraction angiography of the common hepatic artery (right oblique 25°) (**B**) and the Dual-Phase Cone Beam CT ((**C**), delayed phase) confirmed the HCC nodule and vascular anatomy. VR reconstruction obtained from the arterial phase Cone Beam CT dataset (**D**) depicts the correct segment of the artery for microballoon positioning. After balloon inflation (dotted circle), embolization (**E**) was performed with 100 and 200 μm microspheres loaded with 100 mg of epirubicin. CT performed at 1 month ((**F**), arterial phase) showed a complete response. The patient underwent an orthotopic liver transplant 7 months after bTACE.

Recently, a retrospective European multicenter study, led by Golfieri [36], investigated the tumor response rate of bTACE (either DEM-bTACE or c-bTACE) vs. both c-TACE and DEM-TACE using a propensity score matching scheme. Authors reported a similar best target-objective response; however, the complete response at 1–6 months was significantly higher for B-TACE. Finally, the b-TACE cohort showed a significantly lower retreatment rate during the first 6 months. It is worth mentioning that the same group compared TACE with and without balloon occlusion in order to assess in which size range the techniques offered higher complete response (CR) and objective response (OR) rates in a single session, and they concluded that in 30–50 mm HCCs, bTACE showed significant superiority in achieving a CR (72.3 vs. 54.1%) [37] (Figure 2).

These results were also confirmed in a recent European multicenter study [38] that evaluated the long-term (>12 months) oncological outcomes for HCC. The population of the study encompassed seventy-three patients, with HCC of 37 ± 19.9 mm. The complete response rate reported after bTACE was 58.9%, with a median local recurrence-free survival of 31.0 months and a mean overall survival of 50.0 months.

The added value of the employment of a microballoon catheter has relevant clinical implications since, as mentioned above, the main factor influencing overall survival is achieving a complete response, preferably sustained for at least six months, a result that is more difficult to obtain in nodules larger than 3 cm.

## 4. bTACE Technical Notes: How We Do It

In order to perform a bTACE with an optimal pressure gradient effect, some modifications to the standard TACE procedure and angiographic materials should be made (Supplementary Material, Video S1).

Standard and advanced diagnostic imaging (standard digital subtraction angiography (DSA), postero-anterior, right oblique 25°, cone beam CT, and eventually dual-phase acquisition) should be obtained in all patients to enhance the identification of all lesion’ feeders and therefore choose the best site for microballoon placement. The microballoon should be positioned proximal to all feeding vessels in order to maximize its action in optimizing the pressure drop.

After the correct priming of the microballoon catheter (Supplementary Material, Video S2), a coaxial super-selective catheterization is obtained. A diagnostic DSA must be performed to assess the caliber of the feeding vessel to be occluded and, consequently, the target diameter of the inflated microballoon (Figure 2).

The device’s chart indicates the suggested volume to be injected within the microballoon to reach the target diameter. Preferably, inflation should be performed in a straight vascular whenever possible; if not, while positioning the microballoon within a curve segment, operators should remember that this could determine the displacement or jumping of the microballoon. This could easily be handled by gently keeping the microballoon stuck during inflation, preventing dislodgement.

Inflation should be performed under pressure monitoring, which can be obtained by connecting the pressure transducer to the wire’s lumen; this permits us to identify the pressure’s modification at the tip of the microballoon catheter. A BOASP measurement is the only instrument that can help us identify the adequate volume to make the balloon surface adhere to the feeding vessel’s wall, thus preventing over-inflation. The operator should remember that the microballoon is a device that, if over-inflated in a small vessel, can cause damage such as spasms, dissection, or pseudoaneurysm (the latest one was reported as a complication of microballoon employment during the interventional radiologist’s learning curve only in two case series with rates ranging from 1.1% to 2.8% [25,39]). Pressure monitoring should yield to an undulatory curve; though this could not be appreciable if the microballoon catheter is advanced until second- or even third-order branches of the hepatic arteries. In such cases, usually a tremulous curve (lacking a clear systolic peak) is seen. Despite this, evidence monitoring is always able to display a median mmHg pressure. Inflation should be performed by keeping an eye on the pressure monitor to stop inflation as soon as the pressure drop is appreciable. The optimal target of mmHg is around 64 mmHg. As soon as a pressure drop is achieved, inflation should be stopped in order to avoid useless harm to the vessel wall. If the pressure drop is not achieved despite having injected an appropriate volume of solution (according to the proposed table), competitive feeding vessels in the target lesion should be suspected.

Once the appropriate pressure drop has been achieved, the pressure line transducer is removed, and another diagnostic DSA is performed to visualize the obtained redistribution of flow towards the lower-resistance territories (the lesion’s vasculature). This concept is usually appreciable by a better depiction of the lesion’s opacification compared to the surrounding liver parenchyma, if compared with the baseline DSA acquired prior to inflation. If no major differences are appreciable with the baseline DSA acquired prior to inflation, competitive feeding vessels in the target lesion should be suspected.

The embolization is then started according to the preferred embolic material, either drug-eluting microspheres or lipiodol emulsion.

A major difference with the standard TACE procedure is how to assess the correct endpoint of the embolization procedure. Usually during DEM-TACE, embolization is performed until 10-heartbeat stasis is reached. With the usage of the microballoon, by dropping down the “vis a tergo”, this occurrence is not applicable anymore. In our experience, different endpoints were utilized to understand when the embolization procedure was completed:

- Perception of resistance: Even through forced manual injection, no more embolic agent can be injected.
- Reflux despite the presence of the inflated microballoon: Forced injection could determine the overdilation of the vessel wall at the level of the balloon causing reflux.
- Inversion of flow into collaterals: To understand this concept, take a step back to the mechanism of action of the device. Once the balloon is inflated, the restoration of flow beyond the balloon is performed due to the intersegmental collateral opening. This permits the embolic agent to reach the lesion despite the “absence” of the vis a tergo. During the embolization, when the target lesion has been filled with embolics and the pressure within it rises, these collaterals that opened could reverse their flow, pushing our embolics further towards healthy liver segments. Thus, if hepatofugal collateral became appreciable during the embolization, then the embolization should be stopped. Continuous fluoroscopic guidance is mandatory throughout the entire embolization procedure.
- Maximum threshold of drug: The added value of the employment of microballoon catheter has clinical relevance since, as mentioned above, the main factor influencing overall survival is achieving a complete response, preferably sustained for at least six months, a result that is more difficult to obtain in nodules larger than 3 cm.

## 5. Conclusions

The microballoon catheter is a potential game-changer in the field of oncological transarterial therapy, because the catheter is not merely an antireflux device but a liver-flow modifier, obtaining better oncological results in those patients in which usually standard TACE has worse performance.

## Data Availability

No new data were created or analyzed in this study.

## Supplementary Materials

The following supporting information can be downloaded at https://www.mdpi.com/article/doi/s1/: Video S1: Materials needed for bTACE; Video S2: microballoon catheter priming.

## Abbreviations

The following abbreviations are used in this manuscript:

HCC	Hepatocellular Carcinoma;
TACE	Transarterial Chemoembolization;
bTACE	Balloon-occluded TACE;
BCLC	Barcelona Clinic Liver Cancer;
TARE	Transarterial Radio Embolization;
bTARE	Balloon-occluded TARE;
DEM-TACE	Drug-eluting Microsphere TACE;
c-TACE	Conventional TACE;
DSA	Digital Subtracted Angiography;
BOASP	Balloon-occluded Arterial Stump Pressure.
